# Slow Wave Applications of Electromagnetically Induced Transparency in Microstrip Resonator

**DOI:** 10.1038/s41598-018-20771-w

**Published:** 2018-02-05

**Authors:** Muhammad Amin, Rashad Ramzan, Omar Siddiqui

**Affiliations:** 1 College of Engineering, Taibah University, P. O. Box 344, Madinah, Saudi Arabia; 2 Department of Electrical Engineering, UAE University, P. O. Box 15551, Al-Ain, United Arab Emirates

## Abstract

We report a novel guided-wave resonator that supports multiple bands of electromagnetically induced transparency (EIT). The platform for the spatial and spectral interference is obtained by a microstrip transmission line loaded with proximity-coupled open-circuited stubs. We show experimentally that with two microstrip open stubs, a complete destructive interference takes place leading to a single EIT band with near-unity transmission efficiency. More interestingly, the addition of a third stub results in a supplementary EIT band with a Q-factor of 147 and an effective group refractive index of 530. With the open-stub configuration, the EIT phase response can be dynamically controlled by varying the capacitance between the adjacent stubs without breaking the transmission path of the underlying electromagnetic waves. Therefore, the proposed structure is well suited for buffering and tunable phase modulation applications. Since the proposed structures are compact and fully planar, we anticipate seamless integration with low-profile high frequency electronics.

## Introduction

Resonance is a universal phenomenon that deals with the ability of the system to oscillate with greater amplitude at certain frequencies within a spectrum. The suppression of absorption and scattering due to resonance cancellation leads to electromagnetic induced transparency (EIT) along with highly dispersive propagation of transmitted waves^1^. The strong mode coupling leads to drastic “slowing” of light around the EIT band results in enhanced light-matter interactions inside the resonator. The EIT makes the resonant medium transparent to electromagnetic fields within a narrow range of frequencies. Oftentimes, photonic structures supporting single resonance with high Q-factor are used for generating slow light. This reduces the dependence on high input power level required to generate the nonlinear optical effects since the dependence scales with 1/*Q*^2^^2,3^. On the other hand high *Q* factors comes at the cost of reduced bandwidth of operation. Lossless dielectric cavities^4^ or under-damped (lossy) singly resonant plasmonic resonators^5^ have been used for generation of slow light but with a limited bandwidth of operation. One of the ways to overcome this limit is to generate multiple bands of slow light that collectively support broader range of frequencies^6^.

The interference effects leading to sharp spectral features of EIT resonance are ultra-sensitive to surrounding materials. This makes it beneficial for sensing^7–9^ and optical switching^10,11^ applications. It means that the molecular binding events of a biological or chemical analyte within the near field region can be efficiently detected^12^. The highly dispersive EIT effect due to near-field interference can be used to achieve significant enhancement of nonlinear optical processes^13–15^.

While most of the research work on EIT resonances has been undertaken in the fields of Plasmonics and Quantum Mechanics, some recent publications have demonstrated promising potential applications in the classical microwave domain. For example, EIT resonances have been observed in a microstrip transmission line when detuned split ring resonators (SRR) were placed in its vicinity^16^. However, since the EIT was induced as a result of a weaker proximity coupling, unity transmission was not observed which is much desired in slow light and buffering applications. In pursuit of enhanced sensitivities and ultra-high quality factors (Q factors), a microstrip structure was manufactured on a substrate that was made of a two dimensional periodic structure consisting of sub-wavelength dielectric resonators^17^. The multiple Fano resonances were observed due to the interference of the localized Mie resonances with the background Fabry-Perot resonances. Even though high Q-factors with a theoretical limit of 15000 were achieved, the resonator-array substrate rendered a bulky structure hard to integrate within the current trend of low profile electronics. Recently, we demonstrated microstrip based resonator for non-invasive sensing^18^ and an ultrahigh contrast Fano switching applications^10^.

**Figure 1:**
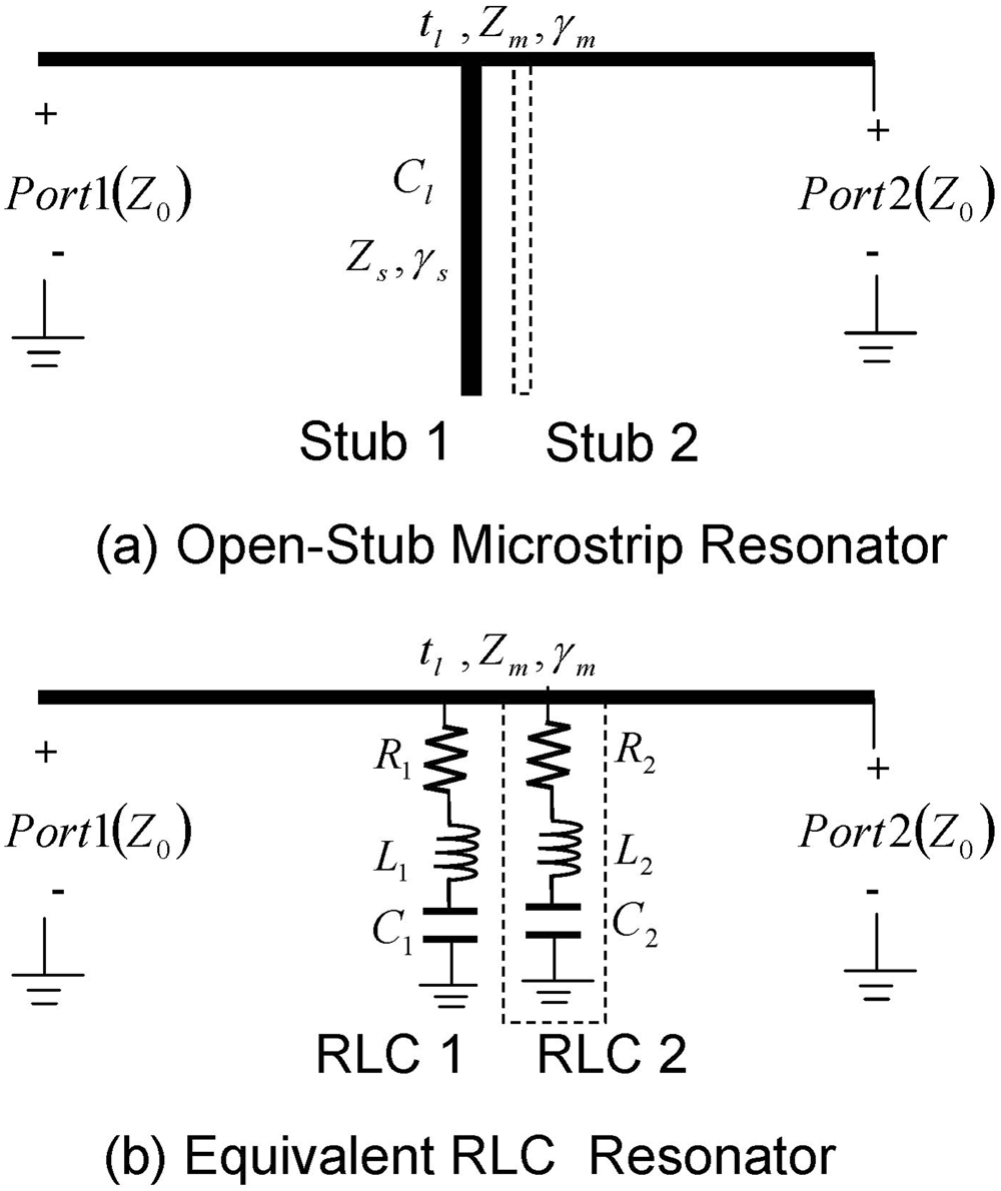
(**a**) The schematic diagram of the proposed multiple open-circuited stubs structure which consist of a microstrip transmission line of length ‘*t*_*l*_’, characteristic impedance “*Z*_*om*_”, and propagation constant *γ*_*m*_ loaded by open circuit stub of impedance *Z*_*s*_, length *C*_*l*_, and a propagation constant *γ*_*s*_ (**b**) The equivalent circuit of the open stub structure consists of series RLC resonators.

In this paper, we proposed a much simpler structure based on microstrip transmission line loaded with closely located identical quarter-wavelength (*λ*/4) open-stubs (see Fig. 1a). The single stub is a transmission-line equivalent of the series RLC resonator connected from the microstrip line to the ground, as depicted in Fig. 1b. However, when identical stubs are connected in proximity, they behave differently from their identical RLC counterparts. The underlying resonance mechanism in the stub configuration stems from the strong mutual interference generated between the electric fields of the slightly displaced adjacent stubs. Therefore, two identical stubs in proximity can be approximated as two slightly detuned resonators leading to extremely narrow band EIT resonances. Unlike the SRR-based microstrip line^16^, the resonating elements in the proposed structure are physically connected to the transmission path. Hence we anticipate stronger dispersive effects resulting in higher Q-factor EIT windows. Furthermore, since the resonance mixing takes place at the open ends of the stubs away from the transmission line, varactors can be conveniently placed without breaking the transmission path for low loss phase-control of EIT applications.

By configuring the proposed structure with multiple open stubs, we show through simulation and experiment that several EIT bands can be obtained through destructive interference of the associated multiple resonances. We also show that with our open-stub structures, we can fabricate novel slow-light applications in microwave domain such as microwave buffers and phase shifters. Note that the EIT response in the proposed structure is characterized by steep changes in both magnitude and phase that are governed by the inter-stub electric fields. Therefore, the dispersion properties can be manipulated by controlling the inter-stub capacitance. Hence, active phase controlled transmission can be potentially realized. Some novel applications based on the microstrip EIT are provided in Section 1.

## Results

### The Microstrip Open-Stub EIT Resonator Design

**Figure 2:**
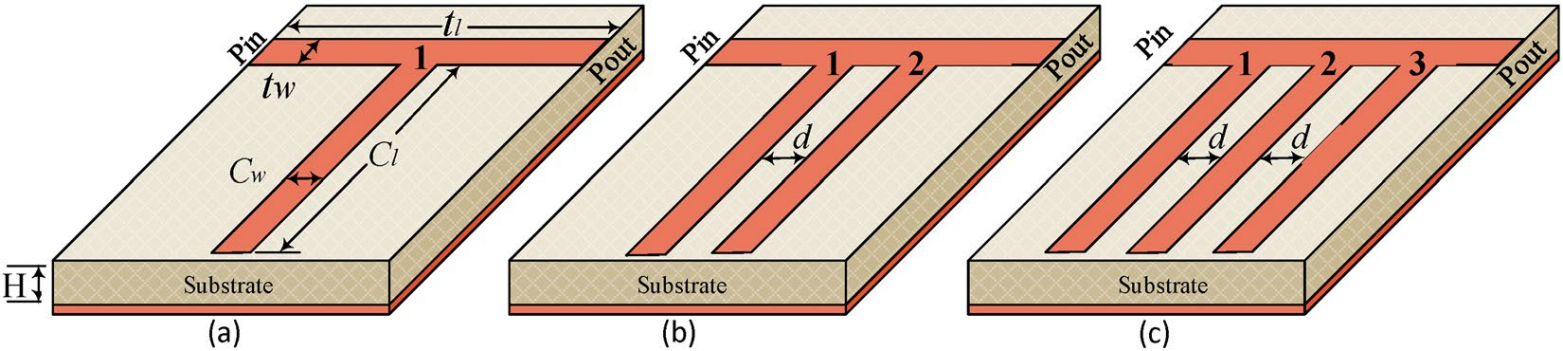
A schematic view of the microstrip resonator is provided. The thickness of substrate is 0.76*mm* with permittivity 2.94. Each resonator consists of open-stub connected to the guiding medium. Three different kind of resonators are considered having (**a**) single, (**b**) double, (**c**) triple open-stubs with dimensions of *C*_*l*_ = 3.5 cm, *C*_*w*_ = 2 mm, *t*_*l*_ = 2 cm, *t*_*w*_ = 3.16 mm, *H* = 0.76 mm and *d* = 2 mm.

From the electric circuit theory view point, a resonance is created in the spectral vicinity of the electric-magnetic energy balance and hence its lineshape can be approximated by frequency response of an equivalent inductive-capacitive (LC) network^19^. Therefore, with circuit representations the classical atomic resonances can be replicated in the lower spectra at macroscopic levels where they can be exploited in more practical microwave and millimeter wave applications^20^. It is well known the Lorentzian lineshape can be approximated by the series RLC resonator connected to a transmission line (Fig. 1b)^21^. A more practical version would be the open circuit quarter-wavelength stub configuration, as shown in Figs 1a and 2a, which are equivalent transmission line implementations of the series RLC resonator^22^. Mathematically, the transmission response can be obtained by applying the microwave Kirchhoff’s current equation to the transmission line and stub combination of Fig. 1 circuit^18,23^,

1 $${S}_{21}=\frac{2{Z}_{o}}{2A{Z}_{o}+\frac{{A}^{2}{Z}_{o}^{2}}{B}+B-\frac{{Z}_{o}^{2}}{B}}$$

where *Z*_*o*_ is the characteristic impedance of the microstrip lines that constitute Fig. 2 structures^24^. The stub configuration has an added advantage that there is an intensive accumulation of the surface charges at the open end. Therefore, the introduction of the interfering resonance is possible by simply adding slightly displaced stubs of similar dimensions to the existing transmission line, as depicted in Fig. 2b,c. Note that with an additional stub, the consequential geometrical asymmetry (stemming from the path difference of the two stubs relative to the input port) and the intense electric fields at the open end lead to the redistribution of the inductive-capacitive behavior of the individual stubs which causes an effective detuning of the frequency response. In other words, the ABCD parameters of Eq. 1 should be reassigned to obtain a close form mathematical expression of the interfering phenomenon that results in the higher order resonances. Due to the complexity and rigor involve in this derivation, the design strategy followed in this paper is to construct the higher order frequency responses by adjusting the position of the stubs by extensive full-wave simulations followed by the experimental verification of the concept. A qualitative discussion of the spectral detuning and the associated distributed (RLC) parameter fitted on the simulated curves will justify the simulation based design procedure, as discussed in Section 3. It is also interesting to note that since the resonant detuning is obtained by proximity effect, a slight change in the mutual coupling between the adjacent stubs would result in spectral shift of the higher order resonances. This real time tunability of the EIT resonance will be further investigated in the following section.

### Full-Wave Simulation Results and the Experimental Evidence

**Figure 3:**
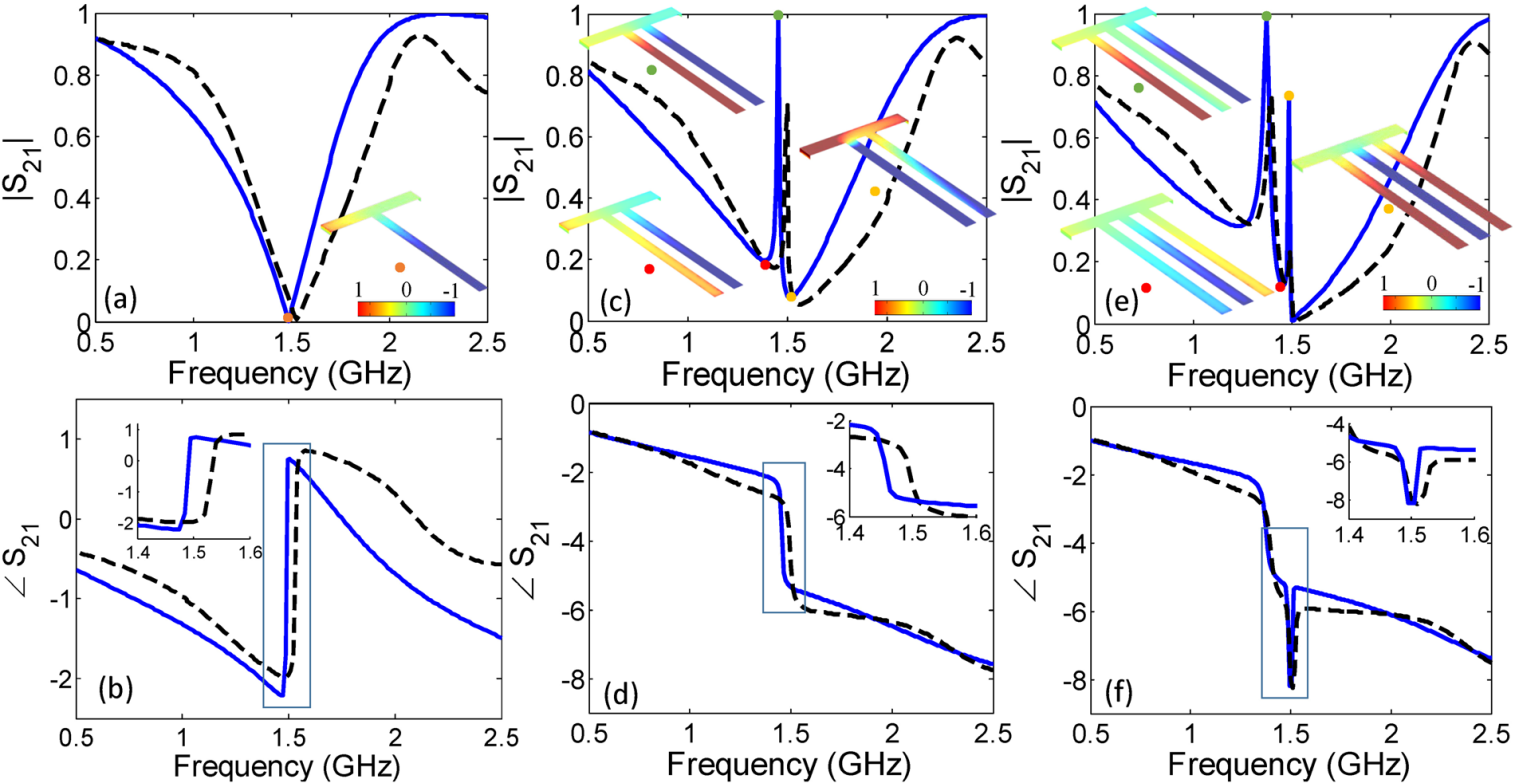
Transmission *S*_21_ characteristics for magnitude and phase of the (**a**,**b**) single, (**c**,**d**) double, and (**e**,**f**) triple open-stub resonator. Simulated results represented in the graph as solid line and experimental results as dashed line. Inset shows surface charge distribution at the resonance point identified as a point on the graph. The surface charge distribution are normalized for negative and positive values.

Consider the single-stub resonator design illustrated in Fig. 2(a) and the relevant amplitude and phase responses plotted in Fig. 3(a) and (b), respectively. As expected, a Lorentzian line shaped resonance is observed around 1.5 GHz in both simulation and experiment. The Lorentzian resonance can be identified by a magnitude dip and an anomalous dispersion regime characterized by the double phase reversal^25^. It should be noted that since the frequency response of any transmission line structure is inherently periodic, the transmittance of the Lorentzian resonator does not remain symmetric farther from its resonance. A slight shift from the simulated resonant frequency observed in the experimental response is due to the permittivity and manufacturing tolerances. Moreover, at the off resonance frequencies, the two responses do not exactly match because of the absence of some fabrication details like effect of connectors and cables which is unaccounted for in the simulations. A steady state solution of the surface charge distribution around the resonance frequency (inset of Fig. 3a) shows an intense accumulation of the negative surface charges on the stub edges as it measures exactly quarter-wavelength. This charge accumulation and the associated intense electric fields provide the platform for the subsequently observed highly selective interference mechanisms.

With the addition of the second identical stub,we expect to achieve two slightly detuned resonances resulting in an EIT window. Looking at the Fig. 3(c), it can be seen that indeed multiple resonances is observed which, in simulation, has a 99% efficient EIT band around 1.455 GHz frequency. There is around a 3 dB loss in experimental results that can be attributed mainly to inherent material losses. The electromagnetic interferences leading to the highly frequency selective resonant response can be explained by considering the steady state surface charge distribution at various points around the resonance. Below the resonance point (1.404 GHz) the second stub carries intense distribution of negative charge compared to first stub which is responsible for the amplitude dip and the subsequent EIT transmission. At EIT resonance frequency i.e., 1.454 GHz the two stubs are completely out of phase leading to a very strong destructive interference between the resonances. Finally, above the resonance frequency i.e., 1.515 GHz the charges at the two stubs gets in phase leading to constructive interference and suppression of transmission. A sharp phase jump can be seen across the EIT band (Fig. 3d) which is expected due to the nature of the narrow band response. The calculated Q-factor in the EIT band is found close to 84.

Similarly, when a third slightly displaced stub is added, the triple resonance mixing produces multiple resonances and as a result two distinct EIT bands are observed around 1.374 GHz and 1.485 GHz frequencies (see Fig. 3(e)). It can be noted that the first EIT band is perfectly transparent up to 99% and second EIT band is 74% efficient. At first resonance point i.e., 1.374 GHz frequency the intensive surface charge distributions on the first and third stubs become out of phase. As a result, the complete destructive interference takes place which is responsible for the first EIT band. Above resonance frequency i.e., 1.454 GHz the charge upon the stubs change phase leading to constructive interference of resonances. Finally, at the second EIT resonance frequency i.e., 1.485 GHz the charges at the two stubs gets in phase between each other, while it remains out of phase with the middle stub. This causes a destructive interference stimulating the second EIT band. It can be noted that even though the second EIT band is less transparent than the first one, it is characterized by much higher charge distributions among the three stubs. Consequently, highly selective (sharp) phase response with a decent Q-factor of 147 are observed which can be exploited in highly sensitive sensing applications.

Figure 3(b) corresponds to phase characteristics of a single stub resonator that exhibits anomalous dispersion around the resonance dip i.e., |*S*_21_| = 0. The anomalous dispersion leads to double phase reversal around resonance frequency. Similarly, Fig. 3(d) corresponds to phase characteristics of double stub resonator, where it can be noted abrupt phase drop of nearly *π* (without phase reversal i.e., (∂∠S_21_/∂*ω*) < 0) appears around the transparency band at 1.455 GHz. This sharp phase change explains the highly dispersive nature and consequently slow group velocity of the transmitted waves through the EIT resonator. Finally, Fig. 3(f) corresponds to phase characteristics of the triple stub resonator, where abrupt phase drop i.e., (∂∠S_21_/∂*ω*) < 0 of nearly *π* appears for both transparency bands around 1.374 GHz and 1.485 GHz frequencies. As amplitude |*S*_21_| hits zero at frequency of 1.5 GHz, the anomalous dispersion effect makes the double phase reversal and therefore appears as groove shape in the phase spectrum.

**Figure 4:**
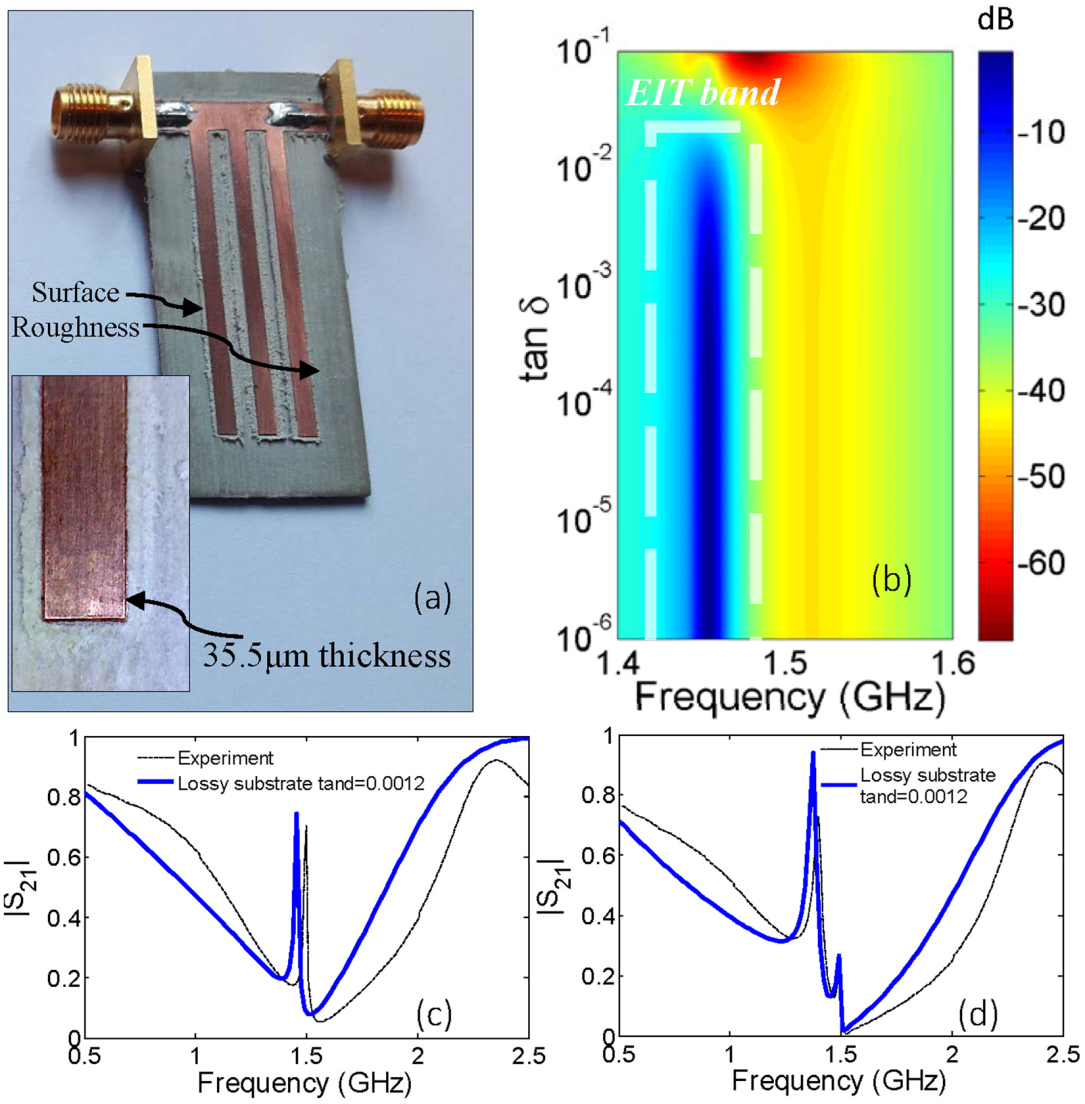
(**a**) Surface roughness of microstrip lines causes unpredictable losses in experiments. (**b**) Simulated results for *S*_21_ as a result of variation in substrate losses (tan *δ*) for double open-stub resonator. It is found that at a loss tangent of 0.0012 leads to an additional losses in the EIT band for the (**c**) the double stub and (**d**) triple stub cases.

It can be noted that the efficiency of EIT does not match well between the simulations and experiments in Fig. 3. The efficiency of EIT band between simulated and experimental |*S*_21_| curves disagrees by approximately 3 dB. The difference can be mainly attributed to the fabrication imperfections and the material losses and can be summarized as follows. Firstly, the leading fabrication imperfection comes from the method of milling that is used in fabrication. As shown in Fig. 4(a), the PCB drilling bit while removing the copper creates rough conducting surfaces leading to transmission losses. Secondly, the simulation setup assumes planar lossless conductive traces. However, in reality, the layer of copper used in the circuit board 35.5 μm thick with a finite conductivity resulting in metallic losses and skin effect which cause further transmission losses. Finally, the effect of the dielectric loss variation on the transmission coefficient is depicted in Fig. 4(b) surface plot. To incorporate these dielectric losses in the EIT circuits, the transmission coefficients are recalculated in COMSOL for a loss tangent of 0.0012 (Rogers 6002) and are depicted in Fig. 4(c) and (d). As anticipated from the surface plot, an approximate 3 dB losses in the double stub EIT band transmission response can be noted. Hence, we can safely assume that the use of precision planar technology (as used in hybrid circuits with conductive materials), low loss and high permittivity substrates, sub-micro meter thickness, and laser milling, the losses related to manufacturing inaccuracies and tolerances can be significantly reduced.

**Figure 5:**
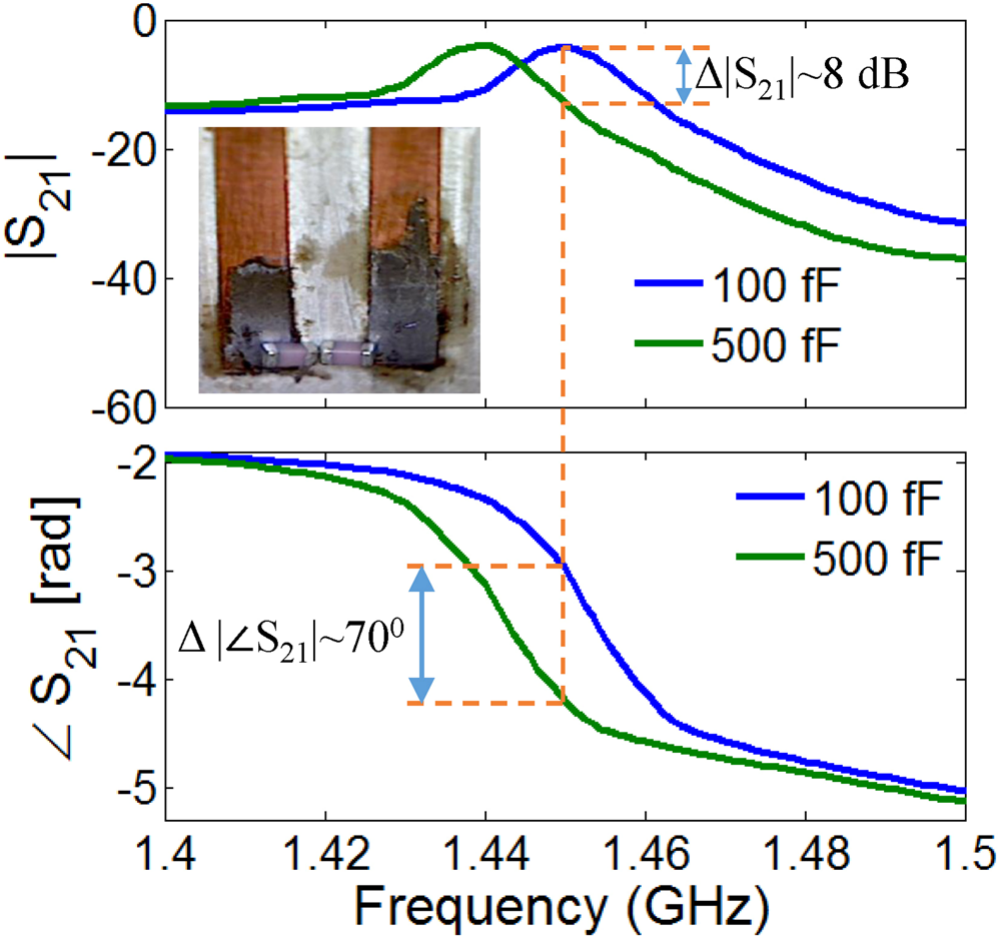
Experimental demonstration the tunability of EIT showing the Transmission Amplitude and Phase responses.

Next, we demonstrate that the EIT band can be tuned to desired frequencies by placing variable capacitors between the open ends of the adjacent stubs. Consider the inset of Fig. 3c, where intense electric fields with opposite polarities are observed in the transparency window. If these strong fields are perturbed by even small capacitance, considerable frequency shift can be anticipated. To show this effect experimentally, two capicitors of values 100 fF and 500 fF are placed sequentially between the adjacent stubs of two-stub EIT structure. Since the exact values were not available, two capacitors were connected in series having double the desired values. As depicted in Fig. 5, the EIT peak shifts from 1.45 GHz to 1.44 GHz, respectively. A very large phase shift of 70^o^ at the first frequency suggests active phase control applications (discussed at a later point in this paper).

To conclude, we provide a summary of the achieved EIT parameters for the three designs in Table 1. With addition of open microstrip stubs and manipulation of their locations, several EIT bands with decent Q-factors and group indexes can be achieved.

**Table 1:** Summary of Resonance Parameters.

Structure	Resonance Type	EIT Quality Factor (Q)	EIT Transmittance Efficiency	Group Index (*ng*)
Single Stub	Lorentzian	—	—	—
Double Stub	Single EIT	85	99%	300
Triple Stub	Double EIT	25/147	99%/75%	160/530

### The RLC Analogues of the EIT Structure

**Figure 6:**
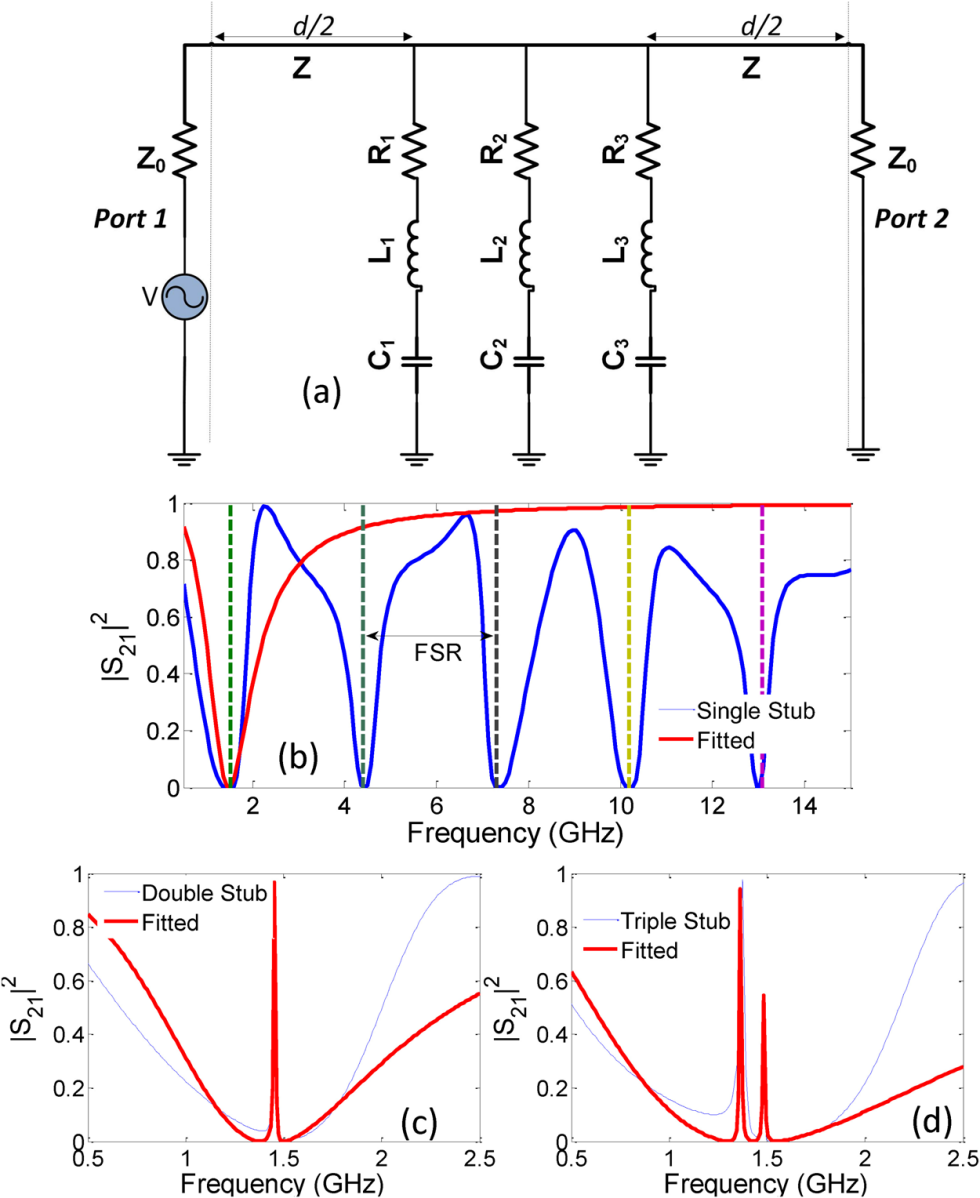
(**a**) Equivalent circuit model for EIT resonator consisting of three connected RLC loops to the main transmission line of length d. Fitted curves for (**b**) single (the resonances repeat periodically at frequency interval given by FSR), (**c**) double, (**d**) triple open-circuited stub resonators.

The EIT windows in a multiple-stub spectral response are the consequences of resonance detuning which results from relative spatial shifts of the open stub locations leading to strong interference effects. As depicted in Fig. 1, different RLC values can be assigned when an additional stub is added to the existing configuration. To represent the detuning effect, the stubs can be replaced by lumped components, as shown in Fig. 6(a) for the three-stub configuration. The transmittance |*S*_21_|^2^ spectra (Fig. 3) can be fitted with the RLC resonator transmission response to subsequently extract the RLC lumped element values. To calculate the transmission response of the RLC equivalent circuit (Eq. 1), first the transfer matrices (ABCD matrices) are generated by the serial multiplication of the individual circuit layers for the single, double, and triple stub configurations:

2 $$\begin{array}{l}{T}_{{\rm{single}}}={T}_{tl}{T}_{s1}{T}_{tl}\\ {T}_{{\rm{double}}}={T}_{tl}{T}_{s1}{T}_{s2}{T}_{tl}\\ {T}_{{\rm{triple}}}={T}_{tl}{T}_{s1}{T}_{s2}{T}_{s3}{T}_{tl}\end{array}$$

where *T*_*tl*_ and *T*_*si*_ are the respective transfer matrices of the host transmission line and the single stub, given by,

3 $${T}_{i}=[\begin{array}{ll}\cos (kd\mathrm{/2)} & iZ\,\sin (kd/\mathrm{2)}\\ \frac{i\,\sin (kd\mathrm{/2)}}{Z} & \cos (kd/\mathrm{2)}\end{array}]$$

4 $${T}_{si}=[\begin{array}{ll}1 & 0\\ {Y}_{i} & 1\end{array}]$$

Here, *k* is the propagation constant, *d* is the length of microstrip transmission line and (*Y*_*i*_) is the single stub admittance, given by,

5 $${Y}_{i}=\frac{1}{1/j\omega {C}_{i}+j\omega {L}_{i}+{R}_{i}}$$

The RLC resonator transmission curves are generated by the nonlinear Levenberg-Marquardt optimization of parameters in Table 2 and are superimposed on the open-stub transmission coefficients in Fig. 6 (b–d). On account of the underlying periodicity, the microstrip open-stubs resonate at every multiple of half-wavelength, known as the free spectral range (FSR). Therefore, the transmittance response of the circuit model does not match with the transmittance of single-stub resonator away from the EIT resonance peaks. In the current design, the FSR is calculated to be 2.88 GHz by calculating the frequency at which the open-stub becomes half-wavelength:

6 $$FSR=\frac{c}{2\sqrt{{\varepsilon }_{r}}{C}_{l}}$$

where *C*_*l*_ is the length of the stub. The extracted RLC values for the three open-stub structures are provided in Table 2. Note the direct correspondence between the single stub resonator and its equivalent RLC model. However, when an identical stub is added, the equivalent lumped parameters of both the stubs attain different values due to the proximity coupling of the intensive electric fields causing the detuning effect. Note that the proximity coupling here refers to the inductive-capacitive coupling between the two stubs that arises due to the small spatial shift of the second stub relative to the input port. It is further discussed under the Discussion part of the paper. In the absence of the proximity coupling, the resonator model would constitute identical lumped component leading to only a single narrow band resonance.

**Table 2:** The fitted RLC parameters generated by the Levenberg-Marquardt Algorithm.

Parameters	Single Stub	Double Stub	Triple Stub
*C*_1_, *L*_1_, *R*_1_	3.33 pF, 3.42 nH, 692 mΩ	1.72 pF, 6.51 nH, 10 mΩ	2.01 pF, 6.16 nH, 0Ω
*C*_2_, *L*_2_, *R*_2_	—	3.01 pF, 4.41 nH, 0Ω	3.48 pF, 4.28 nH, 0Ω
*C*_3_, *L*_3_, *R*_3_	—	—	2.93 pF, 3.62 nH, 92 mΩ

It should be emphasized here that though the underlying detuning mechanism cannot be fully understood from the series RLC equivalence, the model does explain the purpose of arranging the open stub in the spatially displaced geometry i.e. the resonance detuning. Additionally, the use of RLC lumped elements may lead to designing compact wave dispersion based devices based on chip components.

### Suggested Slow Wave Applications

The EIT dispersive properties are utilized for suggesting following slow wave applications for the detuned resonator structure.

### Microwave Buffering

It is often desirable to temporarily buffer RF energy without converting into electrical domain. The EIT resonance is associated with sudden and narrow band phase change which is a desired characteristic for microwave buffers^26^. The ‘slowness’ of a microwave device is measured in terms of the effective group refractive index (*n*_*g*_) which can be extracted from the transmission phase using the well-known relation:

7 $${n}_{g}=-\frac{c}{{t}_{l}}\frac{\partial \phi }{\partial \omega }$$

**Figure 7:**
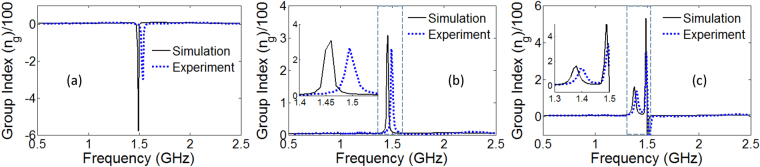
Retrieved values for effective group refractive index *n*_*g*_ for the (**a**) single-stub, (**b**) double-stub resonator and (**c**) triple-stub resonator designs showing the Microwave buffer application of the open stub structures.

Here, *c* is the speed of light, *t*_*l*_ is the length of the transmission path, *ω* is the frequency and *φ* is the phase of transmission coefficient *S*_21_. The simulated and measured retrieved values of the group index for the three open-stub structures are depicted in Fig. 7. Note that for the single-stub resonator, as shown in Fig. 3b, the slope of the transmission phase is positive in the vicinity of the resonance. Consequently, the group refractive index becomes negative (Eq. 7) which refers to an anomalous pulse propagation with a negative group velocity^27^. More interestingly, consider in Fig. 7 b and c the high group refractive index values for the double and triple stub resonators. For the double stub, the simulated value of *n*_*g*_ exceeds 300 within the transparency window. Due to losses in the structure, the experimental group index falls to a modest value of 265. The multiple EIT resonance effect for the triple stub resonator leads to multiple bands supporting slow-light propagation. Around the first EIT band the simulated effective group refractive index reaches 160 and for the second EIT band, it touches 530. The measured group indexes of triple-stub structure attain decent values in the EIT bands, approximately given by 140 and 340. Interestingly, the group index for the triple-stub structure reverses its polarity from positive to negative around the second EIT band. This unusual phenomenon can be utilized as a distinctive spectral signature in sensing applications.

### Active Transmission Phase Control

**Figure 8:**
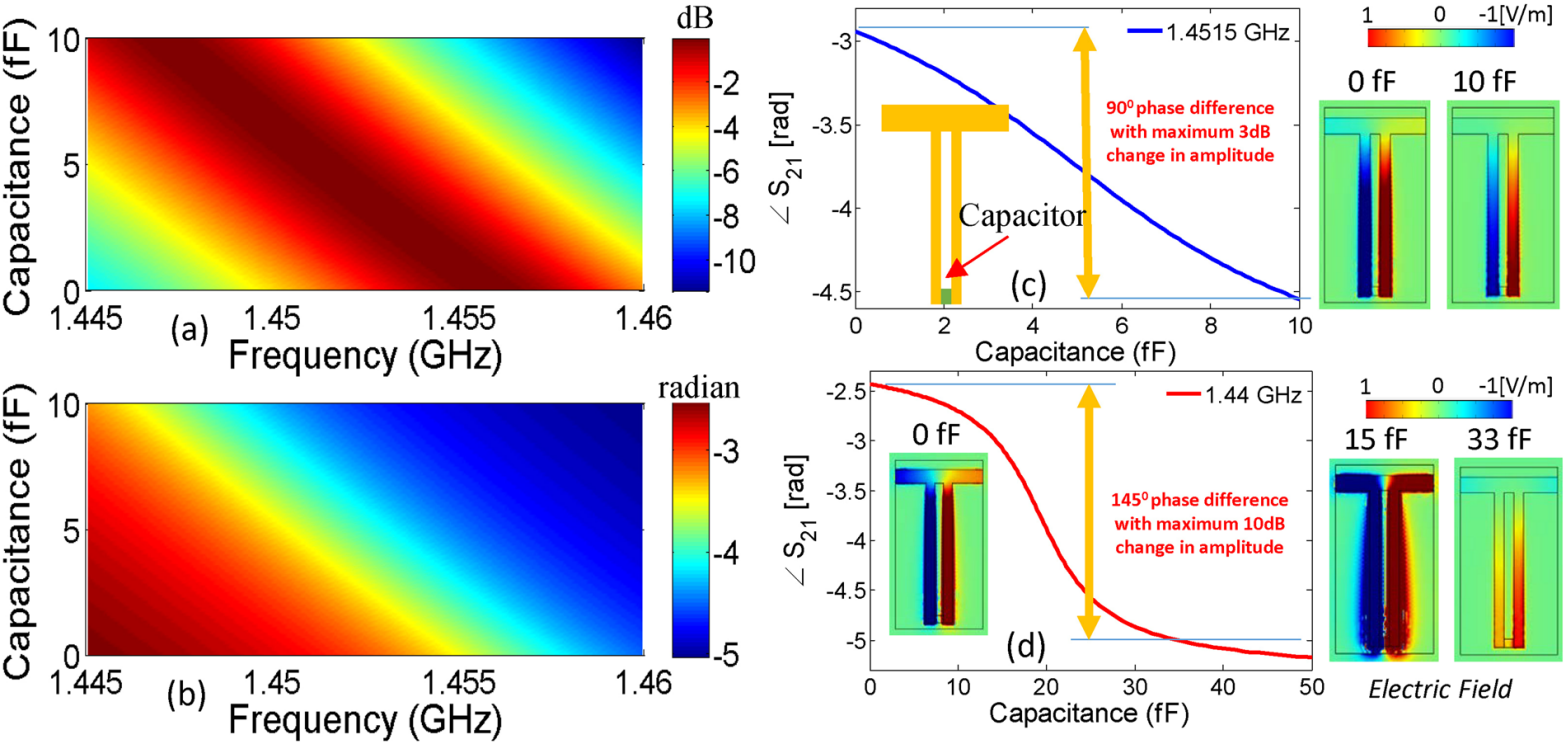
Simulation results for *S*_21_ as result of variation in capacitance between (0 F, 10 fF) at the ends of open stubs (**a**) amplitude variation of *S*_21_ in dB (**b**) phase variation in radians. Phase variation for variation of capacitance between (0 F, 10 fF) for fixed frequencies of (**c**) 1.4515 GHz and (**d**) 1.44 GHz. Electric field distributions at various points are provided in the inset. The colorscale is normalized between maximum positive and negative electric fields.

It is often desirable to actively control the phase of the transmitted signal^28^. The rapid phase change across the EIT resonance can be exploited for achieving this purpose. In order to demonstrate dynamic phase control of transmitted signals in our proposed structure, we numerically insert a small variable capacitive element between the adjacent stubs of the double-stub resonator (see example of tunability in Fig. 5). As it is demonstrated earlier that the electric field is most intense around bottom side of the stubs, the capacitive element is positioned towards the end in between the two stubs (see Fig. 8 inset). The proposed active phase control can be realized by means of adding a varactor element between the two open-stubs.

Due to strong field coupling between the stubs a small capacitance that leads to large red shift in the resonance spectrum, see Fig. 8(a,b). It is shown that a small increase from 0 fF to 10 fF is enough to red shift the EIT resonance by nearly 10 MHz. The corresponding phase change is approximately 90°. This phase control phenomenon is further demonstrated for a fixed frequency of 1.4515 GHz the red shift leads due to increase in capacitance from 0 fF to 10 fF leads to a phase-change of 90^0^, as depicted in Fig. 8(c). It can be noted here that the corresponding change in amplitude is less than 3 dB. To explain the associated resonance phenomenon, the electric field distributions is considered at the frequency of 1.4515 GHz for two different values of capacitance i.e., 0 fF and 10 fF (see the inset of Fig. 8c). The strength of electric field decreases with the increase in capacitance. Subsequently it can be observed that the variation of capacitance between 0 F to 33 fF at a fixed frequency of 1.44 GHz leads to a phase change of 145^0^, as shown in Fig. 8d. The corresponding change in amplitude is less than 10 dB. The electric field distributions at 1.44 GHz is considered for various values of capacitances i.e., 0 fF, 15 fF and 33 fF respectively, as shown in the inset of Fig. 8d. The phase variation is evident from the field reversal on stubs as the capacitance is increased from 0 fF to 33 fF. It is evident that the dynamic change in the phase of transmission can be used in communication systems that utilizes phase shift keying (PSK) modulation scheme. It should be noted that the PSK modulation requires lesser bandwidth compared to other digital modulation schemes. Therefore, inherent high-Q factor supported by narrow EIT bands together with sharp roll-off in the transmission phase are ideally suited for PSK modulation scheme. The EIT resonator with slow wave effect can also be used to provide efficient phase control for Antenna array applications^29,30^.

## Discussion

We showed multiple electromagnetically induced transparency (EIT) bands resonances in a simple multi-stub microstrip structure. The EIT bands were produced due to destructive interference in proximity located open circuit stubs. In particular, we demonstrate that with proper placement of these stubs, near-unity transmission under lossless conditions can be achieved and quality factors and group indexes up to 147 and 530, respectively, can be obtained. The main benefit of the open-stub configuration lies in the fact that the resonance mixing takes place at the open ends of the stubs away from the transmission line. Hence varactors can be conveniently placed without breaking the transmission path for low loss phase-control of EIT applications. We demonstrate a 90^0^ transmission phase shift with a 0 fF to 10 fF capacitance change between the two open-stubs of the resonator. The EIT dispersive properties can be further utilized for developing novel electromagnetic slow-wave interconnects, phase modulators and sensing applications.

Referring again to Fig. 3, the resonance points are identified by the peaks and dips in the transmittance plots. The mechanism of these resonances can be understood by considering the additional phase shift experienced by the transmitted wave when it propagates between two adjacent stubs. In Fig. 2b, the incoming wave reaching the second stub traverses an additional path *d*, which by simple circuit rules translates to the following differential,

8 $${\rm{\Delta }}f={f}_{01}\frac{d}{{C}_{l}+d}$$

**Figure 9:**
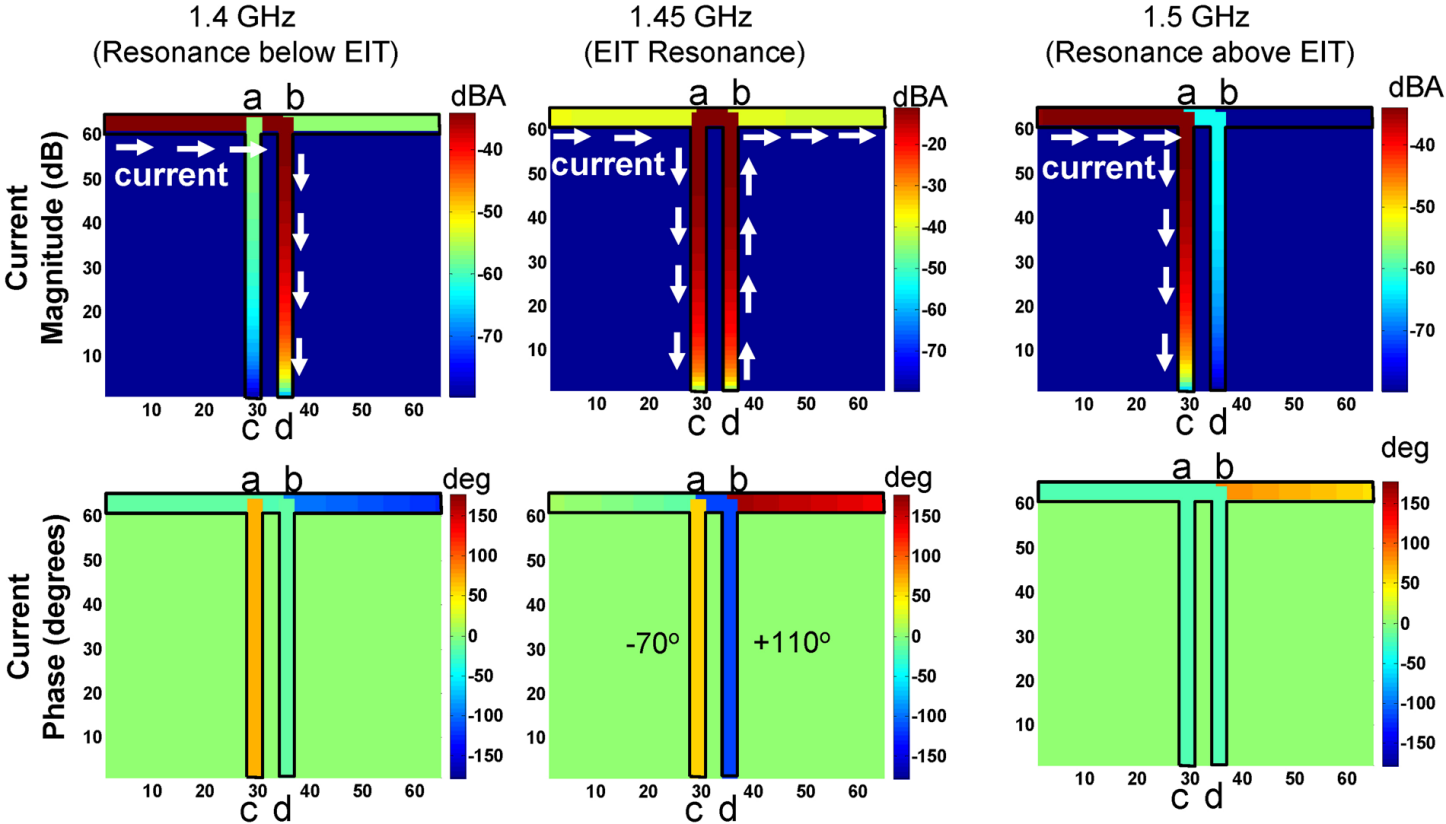
The current magnitude and phase distributions on the EIT double stub structure caluclated from the transmission line analytical model at three spectral resonance points.

**Figure 10:**
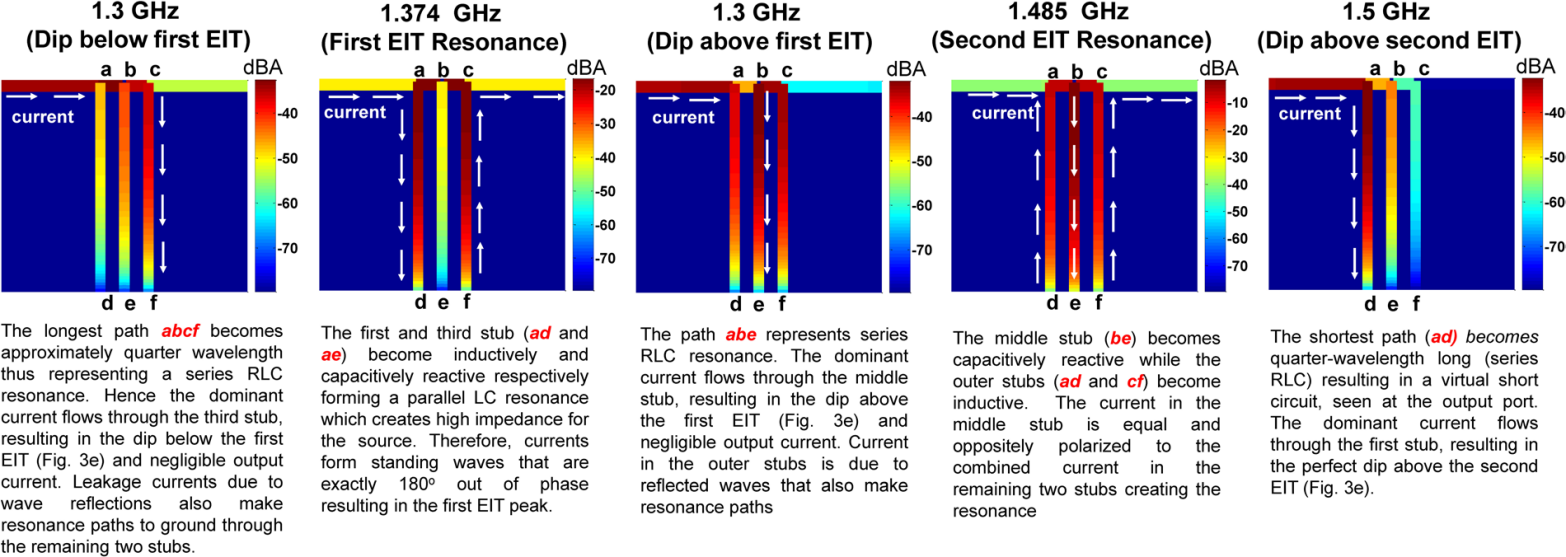
The resonance mechanism in the three-stub EIT structure is explained by plotting the current magnitude distributions at different frequencies corresponding to the dips and peaks of the transmission response.

So if the resonant frequency of the first stub is *f*_01_, the second stub would resonate at a *detuned* frequency of *f*_0_ + Δ*f*. A deeper insight into the resonance mechanism can be obtained by considering the interference effects produced by the detuned stubs that superimpose in the transmission path resulting in characteristic EIT lineshapes. The resonance superposition can be best illustrated by plotting the current distribution on the physical structure at different spectral resonance points applying the Forward Transmission Matrix (FTM) method^23^ for double (in Fig. 9) and triple EIT stub (in Fig. 10) resonators respectively.

**Figure 11:**
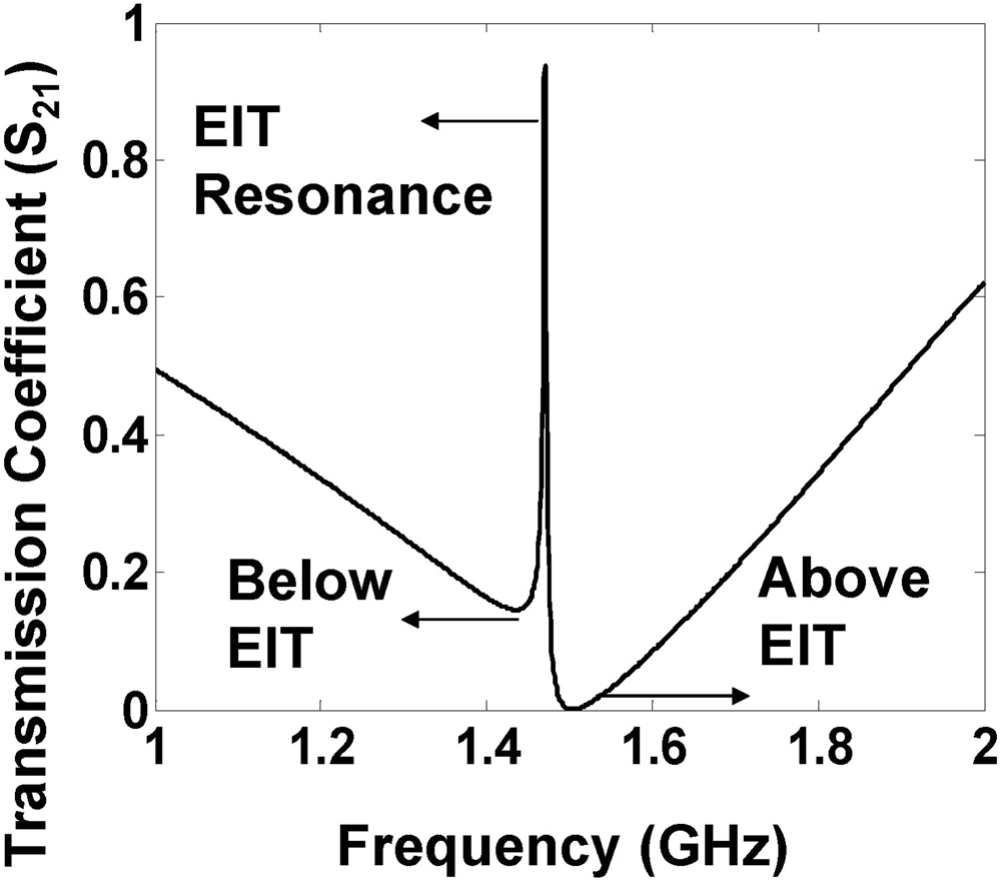
The transmission coefficient of the EIT double stub structure showing the near unity transmission (EIT) and two anti-resonances.

The calculated nodal currents at the three EIT resonances are plotted in Fig. 9 as surface distributions. The first dip below EIT (1.4 GHz) is produced when the conducting path i.e., abd of length *C*_*l*_ + *d* becomes approximately quarter wave-length, thereby representing a series RLC resonator (see Fig. 8). Consequently, like a series resonant circuit, the phase on the resonant branch remains equal to that of the input terminal and the dominant current flows back to the input creating a dip in the transmission. In a similar way, the induced dip above the EIT (1.5 GHz) happens when the first stub attains a length of quarter wavelength. Since there is no alternate path other than the virtual short circuit at point a, all the current returns to the input through the conducting path ac and the output is completely cut-off. As a result, a perfect resonance dip is observed with a zero transmission at 1.5 GHz, see Fig. 11. A more interesting behavior is observed when both the stubs constitute destructively to produce the underlying resonance effect at 1.45 GHz leading to the electromagnetically induced transparency window. During the EIT, the voltages and currents in the individual stubs lead or lag by quadrature-phase, which creates a phase difference of 180^o^. Therefore, the two stubs undergoing EIT can be modeled as a parallel inductive-capacitive (LC) resonant pair which poses a high impedance for the source at resonance. Therefore, as shown in Fig. 9, large standing wave currents that balance each other are produced on the two stubs leading to full power transmission from source to load characterized by a sharp narrow band resonance peak.

The observance of multiple EIT windows in the three-stub structure (Fig. 2c) follows similar principles. To explain these resonance mechanisms, the current distributions for the three-stub EIT structure are calculated and depicted in Fig. 10. Referring to Fig. 3e, a dip in the transmittance magnitude is observed at frequencies where the effective stub length (the actual stub span plus the line segment connected to the wave input) is approximately equal to quarter wavelength. For example, with reference to Fig. 10, the dip at the lowest frequency results from the longest resonance path which consists of the combination of the third stub’s length (*C*_*l*_) and the segment 2*d*. Conversely, a peak is observed when the open stubs are oppositely reactive forming LC pairs which, at resonance, offer high impedance to the source so that the current flows from source to load with 100% power transmission. Consider, in Fig. 10, the difference between the resonance mechanism of the two EIT bands. The first EIT peak which is formed due to the contra-reactive behavior of the two outer stubs and the central stub carries negligible current. On the other hand, all three stubs carry balanced and oppositely polarized reactive currents to form the second EIT resonance peak.

In conclusion, it is shown by analytical modeling, simulation, and measurements that EIT resonance takes place in the microstrip loaded by multiple open-stubs at GHz frequencies. Such microstrip resonators support high group-indexes and high Q-factors for EIT bands along with good transmission efficiency. In depth analysis of underlying detuning mechanism behind EIT is presented through analytical transmission line models and RLC circuit analogues. Slow wave applications of microstrip EIT resonators were discussed for microwave buffer and active phase modulation with minimum transmission losses. Altogether such microstrip EIT resonators have potential applications in phase shifter, buffering, slow wave transmission, and high Q filters.

## Methods

### Forward Transmission Matrix (FTM) Method for Current Distributions

**Figure 12:**
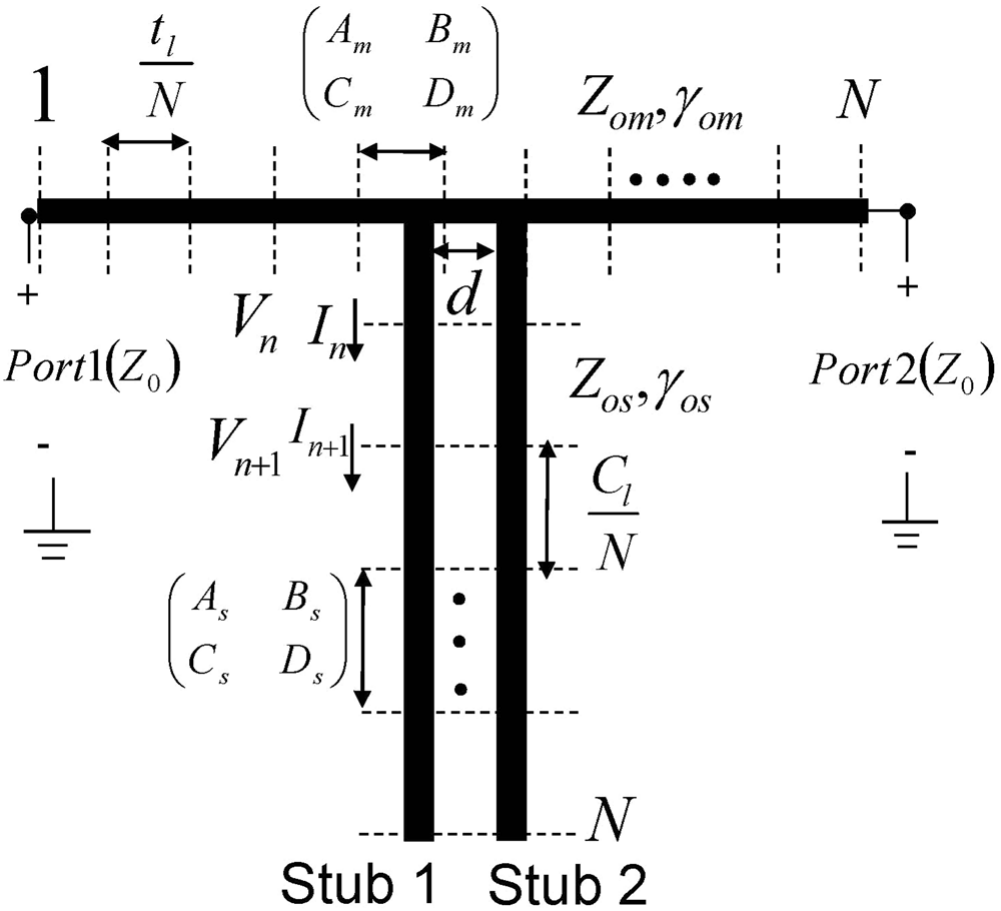
The illustration of the analytical method to calculate the current distribution on the double stub resonator.

The analytical solution of the current distributions on the EIT structure can be obtained by dividing the microstrip and stubs into N differential segments, as depicted in Fig. 12, and applying the Forward Transmission Matrix (FTM) method^23^ to solve the resulting NxN node system. Each differential segment of length *d*_*x*_, characteristic impedance *Z*_*ox*_ and propagation constant *γ*_*x*_ can be represented by the forward transmission matrix:

9 $$[\begin{array}{ll}{A}_{x} & {B}_{x}\\ {C}_{x} & {D}_{x}\end{array}]=[\begin{array}{ll}\cos ({\gamma }_{x}{d}_{x}) & j{Z}_{ox}\,\sin ({\gamma }_{x}{d}_{x})\\ j{Y}_{ox}\,\sin ({\gamma }_{x}{d}_{x}) & \cos ({\gamma }_{x}{d}_{x})\end{array}]$$

where ‘x’ can be substituted with either ‘m’ or ‘s’ to obtain the respective transmission line parameters for the microstrip line or the (open) stub. The differential length *d*_*x*_ = *t*_*l*_/*N* for the microstrip line and *d*_*x*_ = *C*_*l*_/*N* for the open stub. Microwave node analysis^23^ leads to the following system of N simultaneous voltage equations:

10 $${[V]}_{N\times 1}={[F]}_{N\times N}{[{V}_{s}]}_{N\times 1}$$

The simulated transmission coefficient, given in Fig. 11, resembles both full-wave and experimental results. One resonance (peak) and two antiresonances (dips) can be identified in the EIT transmission response. The nodal currents at these resonances *I*_*n*_ can be related to the successive node voltages *V*_*n*_ and *V*_*n* + 1_ in the following manner^23^:

11 $${I}_{n}=\frac{{A}_{x}{V}_{n}-{V}_{n+1}}{{B}_{x}}$$

### The Full-wave Electromagnetic Simulation Method

To observe the resonance formations as a result of constructive and destructive interferences in the proposed microstrip structures, full-wave simulations were carried out by employing the finite-element based electromagnetic simulator COMSOL. Referring again to Fig. 2, the perfect electric conductor (PEC) boundaries were used to model the microstrip lines and the ground plane below the substrate. The input and output port elements were modeled as rectangular surfaces between microstrip and ground planes and the incident electric field were assigned in vertical directions between microstrip and ground plane. The computational domain was terminated by scattering boundary conditions to simulate infinite space surrounding the microstrip circuit.

### The Experiment

**Figure 13:**
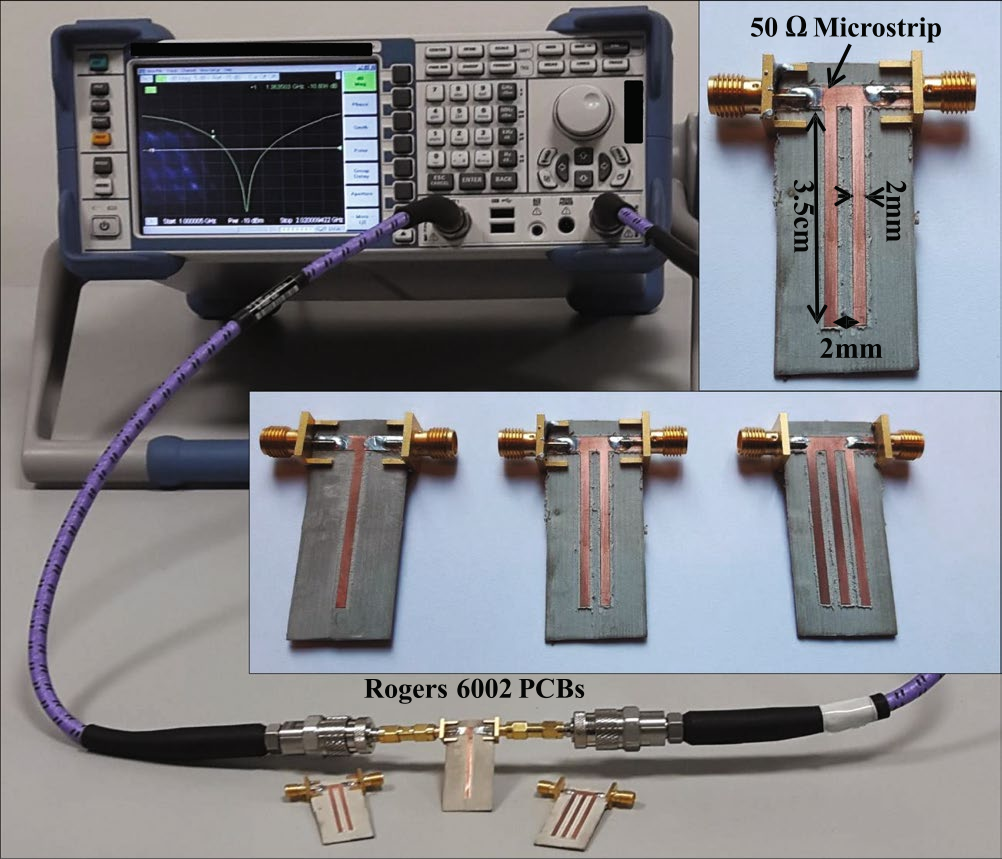
Experimental setup for the characterization of scattering parameters for the microstrip resonators.

For the practical demonstration, the microstrip structures were fabricated on Rogers 6002 substrate (dielectric permittivity 2.94) using MITS AUTOLAB milling machine. The transmission response was measured by the Rohde and Schwarz ZVL13 Vector Network Analyzer. The fabricated circuits along with the measurement set up is depicted in Fig. 13. The simulation and experimental results are provided in Fig. 3.
