# Author Correction: Slow Wave Applications of Electromagnetically Induced Transparency in Microstrip Resonator

**DOI:** 10.1038/s41598-018-24177-6

**Published:** 2018-04-12

**Authors:** Muhammad Amin, Rashad Ramzan, Omar Siddiqui

**Affiliations:** 1 College of Engineering, Taibah University, P. O. Box 344 Madinah, Saudi Arabia; 2 Department of Electrical Engineering, UAE University, P. O. Box 15551 Al-Ain, United Arab Emirates

Correction to: *Scientific Reports* 10.1038/s41598-018-20771-w, published online 05 February 2018

The original version of this Article contained a typographical error in the spelling of the author Rashad Ramzan, which was incorrectly given as Rashid Ramzan. This has now been corrected in the PDF and HTML versions of the Article.

